# Relevance of Electrostatic Charges in Compactness, Aggregation, and Phase Separation of Intrinsically Disordered Proteins

**DOI:** 10.3390/ijms21176208

**Published:** 2020-08-27

**Authors:** Greta Bianchi, Sonia Longhi, Rita Grandori, Stefania Brocca

**Affiliations:** 1 Department of Biotechnology and Biosciences, University of Milano-Bicocca, 20126 Milano, Italy; g.bianchi31@campus.unimib.it (G.B.); rita.grandori@unimib.it (R.G.); 2 Laboratoire Architecture et Fonction des Macromolécules Biologiques (AFMB), Aix-Marseille University and CNRS, UMR 7257, 13288 Marseille, France; sonia.longhi@afmb.univ-mrs.fr

**Keywords:** charge density, fraction of net charge, net charge per residue, charge decoration, linear pattern of charge distribution, charge segregation, polyampholyte, polyelectrolyte

## Abstract

The abundance of intrinsic disorder in the protein realm and its role in a variety of physiological and pathological cellular events have strengthened the interest of the scientific community in understanding the structural and dynamical properties of intrinsically disordered proteins (IDPs) and regions (IDRs). Attempts at rationalizing the general principles underlying both conformational properties and transitions of IDPs/IDRs must consider the abundance of charged residues (Asp, Glu, Lys, and Arg) that typifies these proteins, rendering them assimilable to polyampholytes or polyelectrolytes. Their conformation strongly depends on both the charge density and distribution along the sequence (i.e., charge decoration) as highlighted by recent experimental and theoretical studies that have introduced novel descriptors. Published experimental data are revisited herein in the frame of this formalism, in a new and possibly unitary perspective. The physicochemical properties most directly affected by charge density and distribution are compaction and solubility, which can be described in a relatively simplified way by tools of polymer physics. Dissecting factors controlling such properties could contribute to better understanding complex biological phenomena, such as fibrillation and phase separation. Furthermore, this knowledge is expected to have enormous practical implications for the design, synthesis, and exploitation of bio-derived materials and the control of natural biological processes.

## 1. Introduction

Intrinsically disordered proteins (IDPs) and protein regions (IDRs) escape the paradigm of protein folding and must, rather, be described as conformational ensembles of interconverting conformers. Conformers of biological relevance can be poorly populated and cannot be easily isolated, unless bound to a ligand or partner acting as a conformational stabilizer [1]. The cellular environment (pH, temperature, ionic force, concentration of osmolytes) can influence the relative distribution of such metastable conformers, thereby acting as biochemical sensors and signal transducers [2]. Not surprisingly, IDRs are often located at the protein N- or C-terminus and act as interaction hubs in protein–protein networks [3,4]. This feature is instrumental to IDP/IDR involvement in crucial physiological processes, such as transcription, translation, and cell cycle regulation [2,5,6,7], and underlies the relationships between IDPs/IDRs and diseases (cancer, inflammation, or neurodegeneration) [8,9]. A central challenge for structural biology is understanding how sequence and sequence composition encode structural disorder. Depletion in hydrophobic residues, enrichment in structure-breakers (particularly prolines and glycines), along with polar and charged residues, represent the most common compositional traits and have been employed as diagnostic traits of structural disorder [10,11].

In IDPs/IDRs, the most frequent amino acids with ionizable side chains (i.e., groups that ionize between pH 1 and 14) are Asp and Glu, Lys, and Arg. The ionization behavior is mainly dictated by their equilibrium constant of acid dissociation (the “intrinsic p*K_a_*” value) and their electrostatic environment, including pH. Under physiological conditions of nearly neutral pH, all these residues are charged, although their p*K_a_* can be influenced by several factors, such as dehydration by the Born effect, Coulomb and charge–dipole interactions [12]. Hence, the apparent p*K_a_* can reflect the presence of neighboring peptide bonds, the proximity with other charged groups, hydrogen donors/acceptors, solvent exposition, or burial inside a protein structure, being very sensitive to local conformation [12,13]. Several experimental and computational techniques can be applied to obtain p*K_a_* values of residues embedded in a protein structure. Among these, nuclear magnetic resonance (NMR) spectroscopy allows measurement of the pH dependence of chemical shifts. Such experiments have highlighted that Asp, Glu, and Lys residues in disordered polypeptides, as well as in solvent-exposed regions of ordered proteins [12], have p*K_a_* values close to those measured in random coil models [14], with short- and medium-range interactions prevailing on long-range electrostatic ones [15,16]. Arg represents a peculiar case. On one side, it is highly basic due to charge delocalization; on the other side, the guanidinium group is a very weakly hydrated cation [17]. This latter property facilitates Arg residue burial in hydrophobic micro-environments [18] and its stacking interactions with aromatic protein residues [17,19]. Theoretical studies have modeled IDPs as ideal “charge-decorated” polymers, drawing copiously from polymer physics to describe their peculiar behavior [20,21,22,23,24]. Indeed, in polymer physics, IDPs have been referred to as either polyelectrolytes, with multiple charges of the same sign, or, more frequently (~75% of IDPs), as polyampholytes, carrying both positive and negative charges [25]. The (un)balancing of opposite charges, i.e., the extent of net charge, affects the IDP conformational fate: Electrical neutrality enables polyampholytes to collapse, whereas unbalanced charges result in structural expansion due to repulsive forces [26]. This review focuses on the parameters introduced so far to capture the peculiar electrostatics of IDPs/IDRs and the involvement of electrostatic properties in their physio-pathological roles. In particular, we will discuss how charge density and distribution affect IDP/IDR compactness, aggregation, solubility, fibrillation, and phase separation. Basic information on compaction parameters, fibrillation, and phase separation are presented in three appendices. Describing the mathematical formalisms underlying polypeptide structure goes beyond the aims of this review, which is meant to provide an overview of its practical implications of IDP conformational behavior in the field of protein science and cellular biochemistry.

## 2. From Mean Net Charge to Linear Patterns of Charged Residues

### 2.1. Compositional Classes of IDPs and Phase Diagrams of Protein Conformation

**Figure 1:**
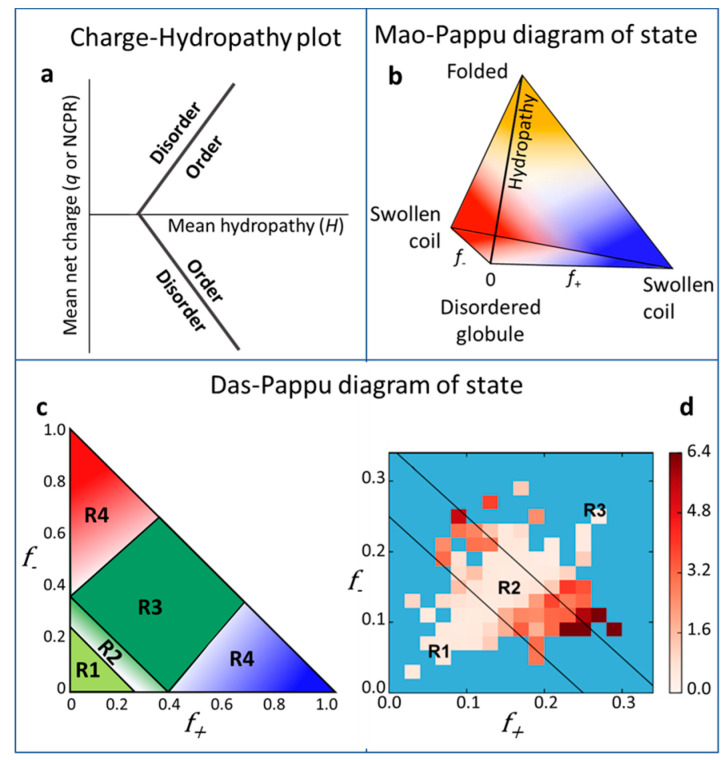
Conformational phase diagrams. (**a**) Uversky’s plot of mean net charge (*q* or NCPR) versus hydropathy (*H*). The two solid lines mark the boundary between disordered and folded proteins [27,28]. (**b**) Mao-Pappu’s phase diagram of conformations for intrinsically disordered proteins (IDPs) and protein regions (IDRs). The three-dimensional sequence space is defined by *f*_+_, *f*_−_, and mean hydropathy. Since high hydropathy and high fractions of charged residues are mutually exclusive, the space is shaped as a pyramid. The yellow area, at the top of the “pyramid”, represents naturally folded proteins, the red and blue regions at the base correspond, respectively, to negatively and positively charged polyelectrolytes (figure inspired by [29]). (**c**) Das-Pappu’s phase diagram of IDP/IDR conformations. The diagram contains four regions (R1-R4) representing distinct conformational classes. R1, weak polyampholytes or weak polyelectrolytes that form globules or tadpole-like conformations. R3, strong polyampholytes that form non-globular conformations, such as coil-like, hairpin-like, or a mixture. R2, continuum of conformations between those in R1 and R3. R4, strong polyelectrolytes with FCR > 0.35 and |NCPR| > 0.3, which sample coil-like conformations approaching the excluded-volume limit [32]. (**d**) Heat map of the protein size distribution predicted by the analytical Ghosh’s model applied to the DisProt entries. The bins correspond to (*f*_+_, *f*_−_) classes. The color scale represents the values of *x*, an expansion index calculated for each protein comparing the ensemble-averaged end-to-end distance predicted by Ghosh’s model with the ensemble-averaged end-to-end distance in the Flory random coil limit, in the absence of any interaction, at T = 300 K. The map represents with color of different intensity the average of *x* values among proteins within a given bin. Blue bins correspond to classes containing less than two proteins. Black lines define R1, R2, and R3 regions as reported in panel C. Reproduced from [33] with the permission of AIP Publishing.

Protein collapsibility is governed by the interplay of intra-chain and chain–solvent interactions. The early concept of an empirical charge-hydropathy (C-H) correlation has been expressed by the so-called Uversky’s plot [27], which classifies IDPs/IDRs according to their position in the two-dimensional space of mean hydrophobicity “H” and mean net absolute charge “*q*”. This latter is equivalent to the absolute value of net charge per residue (|NCPR|), a more recently introduced parameter defined as the difference between the fractions of positively (*f*_+_) and negatively (*f*_−_) charged residues. In the original Uversky’s plot, the line “<*q*> = 2.785 <*H*> −1.151” demarcates the boundary between IDPs/IDRs and natively folded proteins [27], assigning the same coordinates to oppositely charged polymers. To account for the polarity of charged proteins, more recent versions of the C-H plot represent the full range of NCPR values (i.e., not only |NCPR|) with the “*H-q*” space crossed by two “mirror” boundaries (“<*q*> = 2.785 <*H*> −1.151” and “<*q*> = −2.785 <*H*> +1.151”) (Figure 1a) [28]. Still, such plots fail to capture differences that may underlie polymers with similar NCPR values yet endowed with a different number of charged residues. This issue has been illustrated by atomistic simulations and experimental investigations by Pappu’s group on a repertoire of protamines, small arginine-rich nuclear proteins [29]. In spite of their identical NCPR values, these polypeptides possess different dimensions and local conformational preferences. Therefore, with the aim of enhancing Uversky’s C-H phase diagram, the Mao-Pappu’s three-dimensional plot represents the hydrophobicity H on the vertical axis and (*f*_+_) and (*f*_−_) on two horizontal axes, where they vary independently and not cumulatively, as in the case of NCPR (Figure 1b) [29]. Thereby, sequences with low mean hydrophobicity, which initially were collectively considered as “natively unfolded proteins”, are now distinguished into “swollen coils” and “disordered globules”, according to their fractions of charged residues. When *f*_+_ ≫ *f*_−_ and vice versa, which implies large NCPR values, polypeptides can be considered as “polyelectrolytes” and may behave as extended swollen coils. Indeed, the presence of multiple unshielded charges induces chain expansion due to electrostatic repulsions and favorable polymer–solvent interactions, similarly to an ideal polymer in a good solvent [30,31]. On the other hand, when *f*_+_ ≈ *f*_−_, NCPR is close to zero, and polypeptides are “polyampholytes”, which behave as disordered globules governed by attractive interactions. Further, polyampholytes can be classified as “strong” or “weak”, depending on whether they possess a large or small fraction of charged residues (FCR, calculated as the sum of *f*_+_ and *f*_−_) and display specific conformational preferences.

An even better correlation between FCR and IDP conformations is offered by a more recent version of the conformational disorder plot, the so-called Das-Pappu’s phase diagram [11,25,32] (Figure 1c). Herein, low-NCPR IDPs/IDRs are no longer indiscriminately identified as globules, yet they occupy distinct conformational classes—globules, coils, hairpins, chimeras—according to their FCR values. Table 1 lists some examples of proteins belonging to each of these conformational classes. Nevertheless, this classification, as clearly stated by the authors, is valid for IDPs/IDRs of at least 30 residues, with a low overall hydropathy and low proline content [25,32]. Furthermore, it does not provide any insight into how protein dimension varies within these classes [33]. When comparing experimental data with predictions inspired to FCR, or more complex composition-based heuristics, collapsed globules turn out to be less frequent than predicted [33,34,35,36]. Possible reasons for these discrepancies could be searched in the weaknesses of either the experimental or the computational approaches: (i) Collapsed globules have higher aggregation propensity compared to expanded coils, hampering structural characterization at the high protein concentrations required for some biophysical techniques (e.g., NMR, small-angle X-ray scattering (SAXS), etc.); and (ii) the efficiency of prediction algorithms could be hindered by the complexity of the intramolecular interactions’ governing compactness, as well as the interplay with the physicochemical environment [37].

### 2.2. The Concept of Linear Patterning of Charges and Its Parametrization

Asymmetry in electrostatic potentials is a recurrent feature in protein structure, found at the level of the protein backbone [39], secondary-structure elements [40], and supersecondary structure motifs [41]. Herein, we will focus on the effect of the charge distribution and polarization within the protein sequence, considering both the backbone structure and sequence-specific features encoded by the R-groups, i.e., its sequence specificity [37]. In this regard, an important aspect to be considered is the linear charge patterning. Indeed, while theoretical and computational works suggest that weak polyampholytes (i.e., low-FCR proteins) preferentially form globules, strong polyampholytes (i.e., high-FCR proteins) behave very differently from one another, according to the linear distribution of oppositely charged residues in their amino acid sequence [25].

The conformation of high-FCR proteins with an identical charge composition but different segregation of cationic and anionic residues was studied by Srivastava and Muthukumar already in the second half of the 1990s [26]. Monte Carlo (MC) simulations showed substantial differences in the radius of gyration (*R_g_*, defined in Appendix A) between two groups of polymers, in which opposite charges are regularly interspersed or clustered at the two extremities, as a result of the interplay between intrachain electrostatic attractions and repulsions [26]. More recently, the same issue was systematically tackled by either computational simulations or scalable analytical theories, offering a coherent envision, yet using different parameters to quantitatively describe charge patterning. The group of Rohit Pappu has introduced the empirical parameter κ as a measure of the overall charge asymmetry [25]. Upon partitioning the protein sequence into *N* overlapping segments (or blobs, of a size of four to six amino acids, for sequences lacking proline residues), the charge asymmetry of each *i* segment was calculated as:

(1) $$\sigma_{\dot{i}}=\frac{{\left(f_{+}-f_{-}\right)}_{i}^{2}}{{\left(f_{+}+f_{-}\right)}_{i}}$$

The squared deviation of asymmetry was obtained as:

(2) $$\delta =\frac{\sum_{i=1}^{N_{blob}}{\left(\sigma_{i}-\sigma \right)}^{2}}{N_{blob}}$$

Finally, κ was defined as the ratio between *δ* and the maximal value for a given amino acid composition *δ_max_* (κ = *δ/δ_max_*). The minimum value of κ is 0, obtained when opposite charges alternate one by one. The maximum value of κ is 1, accessible to polyampholytes entirely composed of charged residues, when opposite charges are segregated into two clusters. In the seminal Pappu’s work, 30 synthetic variants of a neutral 50-mer (NCPR = 0) were designed to share an identical amino acid composition—(Glu-Lys)_25_—but different κ values (0 ≤ κ ≤ 1), by permutations of oppositely charged residues [25]. The so-called (Glu-Lys)_25_ system of sequences was analyzed using all-atom MC simulations applied to the Flory’s random-coil model [42,43], showing that their ensemble-averaged *R_g_* values inversely correlate with κ [25,32]. Overall, uniformly distributed charges (κ = 0) cause expanded conformations, whereas maximal segregation of oppositely charged residues (κ = 1) results in more compact structures. Calculation of the κ value can be performed for any protein sequence through the web server CIDER (Classification of Intrinsically Disordered Ensemble Regions) (http://pappulab.wustl.edu/CIDER/), developed by Pappu’s lab [11].

Ghosh’s work, instead, tackled the charge decoration issue from a more analytical perspective, introducing a general formalism to describe heteropolymer configurational properties, in the light of sequence specificity [44]. Following the coarse-grained approach introduced by Muthukumar [45], Sawle and Ghosh described pairwise, intra-chain, and short- and long-range interaction forces, taking into account charge patterning by the “sequence charge decoration” parameter (*SCD*), defined as:

(3) $$SCD=1/N\left[\sum_{m=2}^{N}\Sigma_{n=1}^{m-1}q_{m}q_{n}{\left(m-n\right)}^{\frac{1}{2}}\right],$$

where *m* and *n* are the sequence positions within a *N*-mer chain, and *q_m_* and *q_n_* are the residue charges at those coordinates.

**Figure 2:**
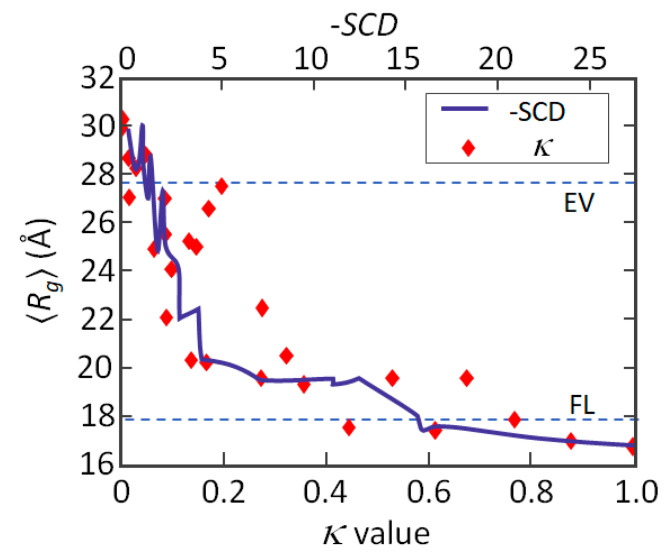
Parametrization of charge patterning. Ensemble-averaged radii of gyration <*R_g_*> for sequence variants of the (Glu-Lys)_25_ system versus κ [25] and *SCD* [44]. The two dashed lines intersect the ordinate at <*R_g_*> values expected for the sequences of the (Glu-Lys)_25_ system, modeled as excluded volume (EV)-limit polymers or as Flory random coils (Flory limit, FL), respectively (figure adapted from [46].

Similarly to κ, the *SCD* value tends to 0 in polypeptide sequences with uniformly distributed opposite charges. Unlike κ, the absolute value of *SCD* increases not only with charge segregation but also with polymer size and is ≤0 (*SCD* = 0 for perfectly alternated positive and negative charges). The correlation between κ and *SCD*, assessed on the (Glu-Lys)_25_ system, is linear, with *R*_2_ = 0.95 (Figure 2). Plotting the simulated *R_g_* values [25] against κ or *SCD* suggests that the correspondence between *R_g_* and *SCD* is even more effective than between *R_g_* and κ, although there is no relationship between Sawle and Ghosh’s formalism and the MC simulations employed for *R_g_* computation. The better performance of *SCD* could depend on the fact that this parameter takes into account all pairwise interactions, regardless of the residue position, while the κ parameter is computed by averaging over stretches of few consecutive charges (blobs) [46]. Nevertheless, Ghosh’s formalism is not well suited to describe collapsed globules (e.g., the model overestimates *R_g_*), possibly due to the employed value of the dielectric constant or to neglecting hydrogen-bonding and ionization equilibria in the model [44,46].

Ghosh’s model has been implemented recently, to account for collapsed globules and coil–globule transitions [33]. Herein, the mathematical formalism relies on minimization of Firman and Ghosh’s free energy (βF), which allows chain conformational properties to be inferred. Moreover, Ghosh’s analytical model was applied to predict the size distribution from the whole DisProt database [47,48,49], revealing significant size differences, even among IDPs with similar FCR values (Firman and Ghosh, 2018). This result is illustrated by a phase diagram, showing the average normalized protein size in the *f*_+_ − *f*_−_ space (Figure 1d). Here, each bin of the heat map corresponds to an (*f*_+_, *f*_−_) class, for which the average value of the chain expansion parameter *x* at 300 K is given. The chain expansion parameter is defined as:

(4) $$x=R_{ee}^{2}∕R_{ee,frc}^{2}$$

where *R_ee_* is the end-to-end distance of the protein of interest and *R_ee,fcr_* is that in the Flory random coil limit (in the absence of any interaction) of the same length. Higher values correspond to darker colors of the scale. The observation that proteins with similar values of *f*_+_ and *f*_−_ are predicted to have different degrees of compactness further confirms that FCR is not per se sufficient to predict chain compactness. Charge decoration, instead, as captured by Ghosh’s model, seems to account for the main sequence determinants of chain conformation.

Moreover, the same analytical model predicts different responses to salt concentration for proteins with similar FCR, highlighting once again the role of charge patterning as a determinant of the conformational behavior of polypeptides [50] (see also below in the next section).

To conclude, according to Pappu’s and Ghosh’s models, charge patterning effectively captures sequence specificity among polypeptide chains that are identical in terms of length, composition, and net charge. In this context, the overall number of charged residues (i.e., FCR) and their pattern seems to act synergistically, and independently of NCPR, to determine conformational properties of polyampholytic IDPs.

## 3. Relevance of Electrostatic Charges in Compaction/Expansion

Charge density and distribution deeply affect conformational states and their transitions, being modulated by pH and salt, as experimentally demonstrated [29,51,52]. The effects of salt in weakening both attractive and repulsive interactions had been well-established previously from a theoretical point of view. According to Debye-Hückel’s theory of charge screening and Higgs and Joanny’s polyampholyte theory [20,53,54], salt addition is expected to produce either conformational expansion within polyampholytes (in which attractive forces are prevalent) or increased compactness within polyelectrolytes (in which repulsive forces prevail). This section provides an overview of experimental investigations depicting the role of charge density and charge patterning on IDPs compactness.

### 3.1. Effects of Charges and Their Screening on Collapse/Expansion Transitions

**Figure 3:**
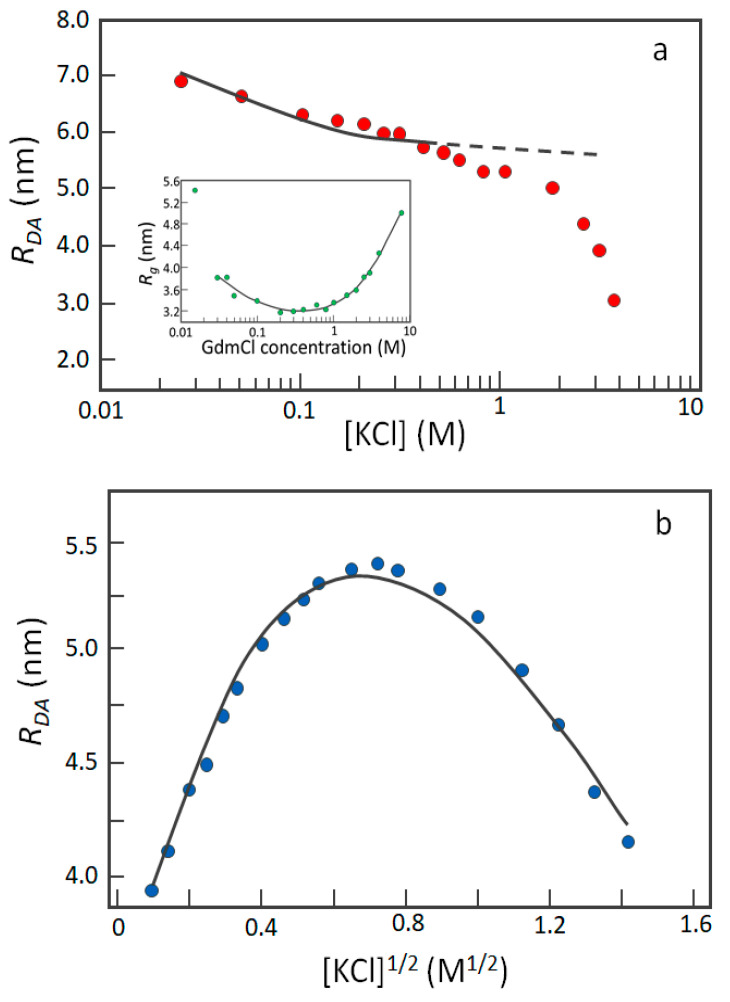
Dependence of IDP compaction on salt concentration. The end-to-end distance obtained from single-molecule Förster resonance energy transfer (smFRET) is expressed as donor-acceptor distances (*R_DA_*) vs. KCl concentration. (**a**) The uniformly charged polyelectrolyte Sic1 undergoes collapse, due to shielding of unbalanced electrostatic charges and attenuation of their repulsive forces. The black solid line represents the fitting by the model described in [55]. At higher KCl concentrations, hydrophobic interactions are likely to overlap with the charge screening effect. The inset shows the behavior of Sic1 *R_g_* exposed to GdmCl. Here, non-chaotropic concentrations (<1 M) cause protein collapse, while higher denaturing concentrations lead to conformational swelling [55], conferring a non-monotonic behavior significantly different from the main plot of panel (**a**). Figures adapted from [55]. (**b**) The polyampholyte Myc undergoes expansion due to the weakening of attractive electrostatic forces at a low KCl concentration (<0.6 M). Here the *R_DA_* is plotted versus [KCl]^½^ to allow fitting of the experimental results by the polyampholyte theory [56]. The dependence of size is modelled on the root square of the ionic strength, which corresponds to the root square of the concentration for a completely dissociated mono-ionic salt. At higher concentrations, the chain collapses again, probably due to the prevalence of hydrophobic interactions over charge shielding. The black solid line represents the fitting by the model described in [56]. Figure adapted from [56].

Charge-mediated conformational transitions rely on both the effective ionization state and solvent exposure of charged residues. The contribution of electrostatics to compactness has been evaluated performing experiments at increasing salt concentrations by single-molecule Förster resonance energy transfer (smFRET), allowing the measurement of molecular distances in the range of 1–10 nm between fluorescence tags in individual proteins. Seminal studies on IDPs have explored the response of polyelectrolytes, such as the C-terminus of ProTα (ProTα, residues 52–111; FCR = 0.700, |NCPR| = 0.533) and the N-terminal domain of HIV-1 integrase (IN, residues 1–56; FCR = 0.267, |NCPR| = 0.067), to 1 M KCl, resulting, respectively, in a 30% and 10% reduction of *R_g_* compared to the absence of salt [51]. The compaction effect, overall ascribable to the attenuation of electrostatic repulsions, depends on the net charge, being more remarkable for stronger polyelectrolytes, according to polyelectrolyte theory [50]. A subsequent systematic study on the N-terminus (residues 1–90) of the *Saccharomyces cerevisiae* cyclin-dependent kinase inhibitor Sic1 (hereafter called “Sic1”) has provided insights into this phenomenon, also suggesting its complexity [55]. Sic1 contains 11 positively charged residue (FCR = 0.122, i.e., 12% of charged residues) and is a weak polyelectrolyte. Figure 3a shows the results of its salt titration monitored by smFRET, with increasing KCl concentrations progressively reducing Sic1 size. Although monotonic, the composite trend of the experimental curve hints to the contribution of several phenomena, which possibly include the different accessibility to the solvent of charged residues and their different response to salt, and the influence of hydrophobic interactions, which prevail upon charge neutralization. Noteworthy, Sic1 undergoes an overall 40% reduction of its *R_g_* in the presence of 1 M KCl. Sic1 compaction is greater than that observed for ProTα (−30%), in spite of a lower |NCPR| (0.122) than that of ProTα (0.533). How to explain the unexpectedly marked compaction of Sic1? Sic1 is a uniformly charged polyelectrolyte (FCR = |NCPR|), while ProTα, as well as IN, are “partial polyampholytes”, as indicated by the non-null difference between their FCR and |NCPR| values. Thus, the strong compaction effect elicited by salt in Sic1 can be explained by repulsion screening and a lack of swelling effects, which likely occurs in ProTα and IN because of the presence of annealed charges. Thus, NCPR and FCR cannot individually explain salt dependence, which is better rationalized by taking into account the balance between attractive and repulsive forces.

When attractive forces are predominant, it is foreseeable that salt induces conformational swelling. Clear examples of this behavior are offered by IDRs belonging to human Myc (residues 353–434), MAX (Myc-associated factor X, residues 22–102), MAD (Mitotic spindle assembly checkpoint protein MAD1, residues 55–136), MLX (Max-like protein X, residues 128–215), and MONDOA (MLX-interacting protein, residues 718–797) [56]. Such highly charged polypeptides (0.3 < FRC < 0.4) behave more markedly as polyampholytes (0.05 < |NCPR| < 0.11), with “annealed” charges conferring compact conformation in the absence of salt. When exposed to low salt concentrations (up to 0.6 M KCl), the screening of attractive interactions causes *R_g_* expansion (Figure 3b) [56]. Above 0.6 M, a chain re-collapse is observed, probably due to hydrophobic interactions, which prevail upon charge screening. Noteworthy, different salts, e.g., LiCl, NaCl, and CsCl, cause compaction to different extents. This salt specificity is reminiscent of the variable salting-out effect along the Hofmeister series and led to the hypothesis that similar factors come into play [56].

For the sake of completeness, it is necessary to mention that several studies on polyelectrolytic IDPs have employed the denaturing salt guanidinium chloride (GdmCl), although its behavior likely reflects the overlapping effects of charge screening, preferential solvation, and chaotropic effects, especially at high concentrations [57].

To conclude, NCPR and FCR are useful parameters to roughly predict whether an IDP behaves as a polyelectrolyte or a polyampholyte in its response to salts, within the general frame provided by Debye-Hückel’s theory, and the polyelectrolyte and the polyampholyte theories. More recently, it has been proposed that chain expansion or compaction induced by salt depends also on charge patterning [50]. An experimental assessment of this theory is given by the different salt sensitivity of protein permutants obtained by simply varying the position of charge residues (“κ variants”) [58] (see Section 3.3). Understanding how environmental conditions affect IDP compactness will contribute to rationalize their function in the cellular context. This knowledge can be exploited also to better control the performance of IDP-based biomaterials and devices [50,59].

### 3.2. Exploitation of Charge Patterning in Stimuli-Sensitive Biopolymers

In the field of material sciences, linear polymers designed for the fabrication of solid surfaces are referred to as “polymer brushes” and have been exploited, for instance, to confer anti-biofouling and anti-frictional properties [60]. IDP-inspired polyampholyte brushes reversibly undergo expansion/collapse transitions in response to external stimuli (i.e., pH, ionic strength, temperature), consistently with their FCR and NCPR [61,62]. IDP brushes profit from a large repertoire of building blocks (i.e., canonical and unnatural amino acids) and, as typical for proteins, offer multiple hierarchical levels of structural organization dependent on their primary structure and post-translational modifications [63]. For these reasons, synthetic IDPs combine the advantages of synthetic polymers and polypeptides.

A fine example of stimuli-sensitive protein brush has been developed by Kumar’s group [59]. A recombinant IDR, rNFH-SA, derived from the heavy subunit of the rat neurofilament complex [64], was grafted in an oriented manner onto a quartz support to functionalize its surface. rNFH-SA is a highly charged polyampholyte, as inferable from its FCR (0.429) and |NCPR| (0.014) values. In addition, our analysis on charge patterning (κ = 0.074) suggests that rNFH-SA may display an expanded conformation. This protein behaves as a polymer brush capable of swelling and collapsing in response to changes in solution pH and ionic strength, in a rather wide dynamic range, not yet fully explained in the light of the theory illustrated in the previous paragraph [59]. Overall, rNFH-SA behaves as qualitatively expected for weak polyelectrolytes, which collapse with increasing salt concentration. A deeper knowledge of polyampholyte electrostatics (charge density and patterning) and of polymer physics could further help in developing “smart biomaterials” with desired properties and capable of complementing the array of already available organic/synthetic polymers.

### 3.3. Effects of Linear Charge Patterning over Protein Compaction

Theoretical studies on charge-decoration and its impact on polyampholyte conformation have been supported by experimental investigations. A plethora of orthogonal biophysical techniques have been employed to explore this dependence. To cite an instance, Tedeschi and collaborators carried out a systematic comparison between three κ-variants for two ~100-residue viral IDPs, merging evidence from SAXS, size-exclusion chromatography (SEC), and limited proteolysis [65]. For each protein, which displays similar values of FCR (~0.3), |NCPR| (<0.05), and κ (~0.2), the authors designed two sequence permutants, shuffling the charged residue positions in order to achieve the highest and lowest possible κ values (average values over the different proteins: κ*_min_*~0.08 and κ*_max_*~0.42) compatible with their natural amino acid composition while keeping the location of non-polar residues unchanged. By doing so, the conformational variability of the variants, relative to the wild-type form, could only be imputable to charge-patterning changes. A direct correlation between κ and protein size was observed. In addition, the study provided hints suggesting that differences in protein responsiveness to charge clustering also reflect differences in proline content (which is indeed different in the two model IDRs considered in that study). In particular, proline residues seem to counteract the compaction effect exerted by charge segregation.

A similar approach, yet enriched in further insights into biological implications, was used by Kriwacki and co-workers and applied to the C-terminal domain of the human cell-cycle inhibitory protein p27^Kip1^ (residues 96–198, hereafter called “p27”), integrating computational simulations and biophysical techniques [66]. The authors kept the primary p27 phosphorylation site (Thr187) unmodified, altering the charge distribution around it, to lower (lowest κ value = 0.14) or increase (highest κ value = 0.78) the κ value relative to the wild-type protein (0.31). Thus, besides the expected κ–*R_g_* inverse correlation, assessed by atomistic simulations and in-bulk conventional techniques (SAXS), the authors could also document differences in phosphorylation efficiency that could be ascribed to sequence-encoded features. Indeed, the efficiency of Thr187 phosphorylation increases with the “local” NCPR of the so-called auxiliary motifs (residues 100–180) [66], highlighting the relevance of linear charge patterns in supporting (or contrasting) a primary physiological function.

An even finer, yet consistent, characterization of p27 sequence permutants (κ values of 0.14 and 0.56) was performed by Barran’s group, exploiting native mass spectrometry coupled to ion mobility, a valuable technique to interrogate IDP/IDP ensemble conformational heterogeneity [67]. Collisional cross-sections of the permutants proved that charge patterning dramatically affects IDP/IDR compactness, with the high-κ variant displaying a lower conformational heterogeneity, compared to the wild-type and low-κ variant [58]. The latter turned out to be insensitive to increasing salt concentrations, whereas the high-κ variant displayed a conformational expansion at high ionic strength [58].

Other insights into the functional relevance of charge patterning have been obtained for RAM (RBP-Jk-associated-molecule) region, a 111-residue IDR belonging to the intracellular domain of the Notch receptor (NICD) and involved in a transmembrane cell-to-cell pathway controlling cellular differentiation and stem-cell fate [68]. The limited dispersion of κ values among distantly related RAM orthologues led to lay the hypothesis that its charge patterning responds to a functional requirement, i.e., mediating its binding affinity for CSL, an element of the tertiary complex (NICD-CSL-MAML) involved in Notch activation. Among RAM charge permutants, it was observed that *R_g_* and an affinity for CSL decrease with increasing charge segregation, causing a significant loss in Notch transcriptional activation. Thus, experimental and computational data consistently suggest that charge decoration influences the conformational preferences of IDPs and can be considered as an evolutionary-selected trait of crucial importance for their functions.

## 4. Relevance of Electrostatic Charges in Protein Solubility/Aggregation and Fibrillation

Protein solubility corresponds to the ability of a polypeptide chain to dissolve into a solution, notably aqueous, and is governed by the competition among chain–solvent, inter-chain, and chain–chain interactions. Such labile equilibrium is severely impacted by solvent, ionic strength, temperature, and pH. Typically, at pH values higher or lower than the protein pI, protein–protein interactions are disfavored, in favor of chain–solvent ones, therefore increasing its solubility. Recent studies suggest that the correlation between protein pI and the pH of their (sub)cellular environment is simply a neutral “by-product” of the main adaptive selection aimed, instead, at favoring structural metastability [69]. In analogy with this original view, it could be hypothesized that protein solubility is the result of a trade-off between metastability and biological activity.

The issue of protein solubility has been widely addressed by physical statistics, considering the polymer–solvent interaction parameter *χ* [70,71] and polymer–solvent interaction energy. Briefly, *χ* can be considered as a measure of solvation enthalpy, namely the enthalpy associated with transferring the polymer from the gas phase into water. Therefore, a distinction can be drawn between “good” (*χ* < 0) and “poor” (*χ* > 0) solvents, regarding the ability to solvate a given polymer. Polymers expand and dissolve in a good solvent, while they collapse in a poor one. Referring to polypeptide chains, the propensity to be solvated depends on both backbone and side chains. Although in aqueous media the protein backbone is prone to collapse, it is the interplay among the sidechains, backbone, and solvent that decides the actual solvation fate of a protein and could support or reverse the intrinsic backbone-compaction propensity [37]. Thus, sequence specificity could account for the divalent nature of water, being a poor solvent for globular folded proteins and a good one for IDPs [37].

A plethora of predictive programs have been developed to infer aggregation propensity from the primary structure [72,73]. The next section will focus on the role of electrostatic charges in determining IDP solubility and aggregation properties.

### 4.1. Effects of Charge Density on Protein Solubility/Aggregation

The effect of electrostatic charges on protein solubility is controversial. Modulating protein conformation and solvation through the manipulation of pH-sensitive groups represents an exciting challenge [74,75,76], limited in practice by the difficulty of producing well-folded charge variants of globular proteins. The high designability of IDPs [77], herein meant as the number of sequences encoding conformational ensembles of similar compaction properties, is exploitable to conceive synthetic solubility-enhancing tags [78]. Solubility-enhancing tags can promote solubilization through a dual mechanism: (i) By increasing the relative proportion of solubility-enhancing amino acids with respect to the overall residue composition of the fusion construct [79]; and (ii) by acting as “entropic bristles” (EBs) through random movements around their point of attachment. EBs entropically exclude the contact with large particles, i.e., other proteins/peptides, thus reducing the probability of the fusion protein to undergo aggregation, without excluding small molecules, such as water, salts, metals, or cofactors, which in fact increase solubility [80].

A first effort to prove IDPs as effective solubility tags was performed by Santner and collaborators, who compared the solubilization performances of four synthetic intrinsically disordered tags to those of several well-established folded tags [81]. In this pivotal work, polyelectrolytes of different lengths (60, 144, 250 residues) and net charge (−24, −25, −41, and −65) yet similar pI (from 2.5 to 3) were tested. Interestingly, the chain length turned out to be more crucial than the sequence composition, with larger proteins being more effective EBs [81]. It should be emphasized that the proteins selected in this study, although presenting diverse net charges, have a seemingly high charge density, i.e., NCPR values (~−0.40; −0.29; −0.26). Therefore, the chain length, rather than the charge density, represents the most significantly diverse parameter among the analyzed proteins, without ruling out any contribution of charge density itself.

This issue was more directly addressed through a set of synthetic IDPs derived from the N-terminus moiety of measles virus phosphoprotein (PNT, 230 residues) [82], whose sequence naturally possesses an acidic pI (4.88) and an almost balanced set of oppositely charged residues, thus resulting in an NCPR ~0 (−0.071). PNT synthetic variants have the same length, FCR (0.257 ± 0.004), and hydropathy score (3.826 ± 0.067), yet they display different NCPR values (from −0.248 to +0.216) and therefore different pIs (from 3.37–9.61) [83].

As expected, each synthetic protein experiences a solubility loss at its pI. Furthermore, the “aggregation intensity”, namely the proportion of insoluble protein, turned out to be directly correlated to |NCPR|, with low-NCPR proteins remaining mostly soluble and almost aggregation free, independently of pH. PNT variants more responsive to pH are able to “transmit” their aggregation propensity to resilient proteins, such as green fluorescent protein, embedded in the same chimeric construct [83]. Overall, these observations may contribute to understanding the behavior of IDPs in response to events affecting protein NCPR (i.e., post-translational modifications, mutations, environmental changes). For instance, it could be argued that high-NCPR proteins (i.e., polyelectrolytic IDPs) are much more sensitive than polyampholytes to even slight pH changes. Results from [83] have led to the development of an empirical equation suitable to predict pH-dependent aggregation of amyloidogenic IDPs and, hence, to promote the design of synthetic solubility/aggregation tags, as well as reversibly aggregating nanofibrillar materials [84].

### 4.2. Relevance of Electrostatic Charges on Fibrillation

Amyloid fibrils have been associated with important biological functions [85,86] and a plethora of pathologies, including socially relevant neurodegenerative diseases, such as Alzheimer’s and Parkinson’s diseases [87]. Aggregation can be triggered in proteins, as the result of either “ordering” of disordered regions or “disordering” of well-folded structures [88]. For a more detailed description of fibril structure, see Appendix B. The research on the “amylome”, the ensemble of amyloidogenic proteins in a proteome, has indicated that most proteins can form amyloid fibrils in vivo [89], with the involvement of different sequence features. Most frequently, canonical amyloid fibrils are encoded by stretches of 5–15 residues of aliphatic and aromatic amino acids (Riek, 2018). Nevertheless, the aggregation of prions and prion-like domains is apparently independent of aliphatic residues and influenced by pH [90,91,92]. In this subclass of amyloids, which are self-perpetuating and infectious, sequence determinants have been elusive for years, since cryptically encoded by rather long (at least 60 residues) and disordered sequences, containing a few amino acid types (i.e., low-complexity regions) rich of Tyr, Gly, and polar residues (mostly Gln and Asn) [90,93,94,95], which argue for the involvement of hydrogen bonds, van der Waals, and π–π stacking interactions [96,97,98]. Short linear sequence motifs rich in polar residues (e.g., SYSGYS from human FUS protein, or GNNQQNY from yeast Sup35) have also been recognized as “nucleation centers” or “soft-amyloid cores”, effectively promoting both spontaneous and seeded aggregation in proteins [99,100], and natural and synthetic peptides [101,102,103].

The structures of human prion protein (PrP) fibrils, recently solved by Cryo-electron microscopy (Cryo-EM), have clarified the role of hydrophobic, polar, and charged residues [104,105]. The fibrils obtained from residues 23–231 of human PrP display a hydrophobic and compact core stabilized by an intramolecular disulfide bond (between Cys179 and Cys214), while a mostly hydrophilic surface is exposed to the exterior. Indeed, each fibril consists of two protofibrils intertwined in a left-handed helix, with Lys194 and Glu196 from opposing subunits forming salt bridges and creating a hydrophilic cavity at the interface of the two protofibrils [105]. This structure is coherent with previous data suggesting the critical role of pH in promoting prion fibrillization [92].

**Figure 4:**
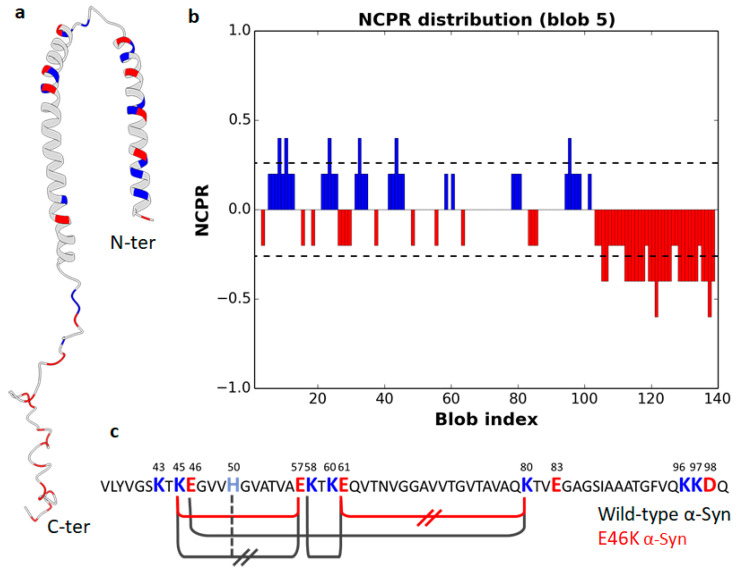
Charge density and distribution of α-Syn. (**a**) Cartoon representation of an NMR structure of micelle-bound human α-Syn (PDB 1XQ8; [118]). Image created with UCSF Chimera [119]. (**b**) NCPR profile along the linear sequence of α-Syn. The blue and red peaks denote positive and negative charges, respectively (plot obtained by CIDER, [11]). (**c**) Primary sequence of the wild-type α-Syn fibril core (aa 37–99). Charged residues are shown in colored bold letters and those interacting in the structures of acetylated wild-type (PDB 6A6B; [120]) and E46K (PDB 6L4S; [121]) α-Syn fibrils are connected by black and red solid lines, respectively. Intermolecular interactions are marked by transverse parallel lines. Figure adapted from [121].

As concerning canonical amyloid fibrils, polar and charged residues are frequently arranged in disordered protrusions at the fibril edges of the hydrophobic fibril core, being functional to elongation through transient interactions with incoming monomers [106]. Such flexible charged structures have been detected, for instance, in the aggregation products of α-synuclein, Aß peptide, and tau protein [106]. Nonetheless, electrostatic interactions can also play a direct role stabilizing the cross-β-sheet structure. In this respect, we summarize here available data on the impact of charge decoration and electrostatic networks in α-synuclein (α-Syn) fibrillation, generally regarded as the pathological hallmark of Parkinson’s disease [107,108] and other synucleinopathies [109,110]. Charge density and, ante litteram, charge segregation of α-Syn have been deeply investigated for their effects on fibrillation. α-Syn is an IDP composed of 140 residues (FCR = 0.279; NCPR = −0.064; κ = 0.172) containing an N-terminal domain with a highly conserved α-helical-prone lipid-binding region (residues 1–60), a central hydrophobic region (NAC) essential for α-Syn aggregation (residues 61–95). and a C-terminal tail (residues 96–140), acting as an interaction hub for several proteins [111,112,113,114,115] (Figure 4a). The NCPR profile calculated by CIDER (Figure 4b) suggests a sharper distinction between an amphipathic and amphoteric N-terminal moiety (residues 1–102), in which opposite charges alternate almost regularly (κ = 0.082; FCR = 0.311), and a highly acidic C-terminus (residues 103–140) with highly clustered negative residues (FCR = −0.368). In vitro fibrillation of α-Syn is markedly pH dependent and occurs at higher rates at acidic pH [116,117]. This acidification effect can be explained considering the α-Syn domain structure. Indeed, acidification increases the net charge of the N-terminal moiety (from +6 at neutral pH to +17 at pH 3) and neutralizes the negative charge of the C-terminal domain (from −15 at neutral pH to 0 at pH 3). Albeit this transition involves a similar number of charged residues in each protein moiety (+17 at the N-terminus and −15 at the C-terminus), the N-terminal region at an acidic pH displays a markedly lower charge density (local NCPR_1–102_ = +0.17) than the C-terminal domain at neutral pH (local NCPR_103–140_ = −0.39). Such a reduction in charge density weakens intra- and inter-molecular electrostatic repulsions, as well as solvent interactions, and ultimately enhances hydrophobic interactions responsible for fibrillation [116,117].

The role of the C-terminal tail in pH-driven fibrillation of α-Syn has been further supported by experimental [122] and computational studies [123]. Data reported so far depict the C-terminal domain as an effective EB. As long as it is highly charged, expanded. and capable of large conformational fluctuations, it acts as an intramolecular chaperone, counteracting aggregation. The N-terminal region, instead, populates an ensemble of unfolded conformations with some intrinsic helical propensity, in equilibrium with membrane-bound, monomeric and multimeric, and helical structures [118,124,125] (Figure 4a). Cryo-EM studies indicate that the fibril core of α-Syn consists of residues 37–99, while the N- and C-terminus remain flexible and not resolved. The structural detail of fibrils indicates the key role of a network of electrostatic interactions involving intramolecular (i.e., E46-K80, K58-E61) and intermolecular salt bridges (K45-E57) (Figure 4c) [120]. The analysis of the E46K variant, associated with a severe form of familial Parkinson’s disease, highlights the importance of electrostatic interactions in defining the fibril morphology, as well. Indeed, with respect to the wild type, the E46K variant reshapes the above-mentioned electrostatic network, and forms a smaller fibril core (residues 45–99) and a distinct fold. This is of utmost relevance with regard to the pathogenic mechanism, as E46K fibrils are less resistant to proteases and mechanical stress and, therefore, more prone to propagation [121].

As expected from a role of electrostatic interactions in fibrillation, salts affect the aggregation kinetics of amyloid proteins. However, they do that in a highly complex way. A systematic analysis of the effect of salts on protein aggregation kinetics has been performed by testing ions from the Hofmeister series in real-time quaking-induced conversion assays [126]. This study reveals different effects of ions, depending on their position in the Hofmeister series, in line with a crucial role of protein hydration in fibril formation [127]. The effect is more remarkable for anions than cations and dependent on the biological matrix. Furthermore, the dependence of aggregation kinetics on Hofmeister ions is highly protein specific. The PrP and tau have opposite trends along the series, while α-Syn displays a bimodal response, with enhanced kinetics at both ends of the series. These results are in line with a complex interplay of electrostatic, hydrophobic, and hydration effects governing protein fibrillation and with the involvement of specific residues of different nature in these proteins.

## 5. Relevance of Charge Decoration in Phase Separation

Spatio-temporal control of intracellular reactions is based on a finely regulated molecular trafficking through cellular compartments. Besides membrane-limited structures, membrane-less organelles [128,129,130] contribute to compartmentalization, separating molecules by liquid demixing and confining them in droplets at higher local concentrations than the surrounding matrix [131,132], thereby creating dynamic proteinaceous microreactors [133] (see Appendix C). LLPS can occur through heterotypic (i.e., interaction and de-mixing of two or more polymers) or homotypic coacervation (i.e., single-polymer self-association) [134,135]. Coacervation usually occurs at concentrations and temperatures thermodynamically favoring self-interaction of polymers. Biomolecular condensates are generally enriched with multivalent molecules prone to establishing multiple intra-chain and interchain interactions [128]. Therefore, proteins with a modular architecture, encompassing low-complexity regions and/or repeats of short-linear motifs, are particularly well suited to nucleate coacervation. It is therefore not surprising that IDPs/IDRs display a strong propensity to undergo LLPS.

Different types of non-covalent interactions have been implicated as triggering factors: Hydrogen bonding, cation–π contacts, electrostatic and dipolar attractions, and π–π interactions between aromatic rings [128,136] (see also Appendix C). Our understanding of sequence determinants of phase behavior is in its infancy and we have just started learning its “grammar” [136,137]. Aromatic residues (Phe, Tyr), along with charged ones (Arg in particular), have often been shown to be key determinants of in vivo and in vitro LLPS occurring through short-range attractive forces due to π–π or cation–π interactions [134,136,138,139,140,141,142]. Fewer studies have investigated long-range electrostatic interactions between charged residues and their patterning on LLPS [46,139,142], although they might have a prominent role, due to the compositional features of IDPs [143]. To assess the role of electrostatic interactions, experiments are typically carried out in the presence of varying concentrations of salt, most often represented by NaCl. A seminal work on the N-terminal domain of Ddx4 (Ddx4^N^), a primary constituent of human germ granules, has unveiled that its coacervation is dominated by π–cation interactions involving Phe and Arg residues. The LLPS of Ddx4N is hence extremely sensitive to ionic force, as highlighted through experiments at increasing concentrations of NaCl [134].

Two other illustrative examples are provided by hnRNPDL and NPM1, which are involved in a form of muscular dystrophy and in the spatial organization of the nucleolus, respectively. HnRNPDL (and its isoforms) was found to only form liquid-like droplets at low ionic strength [144], and homotypic LLPS of NPM1 is similarly impaired at high NaCl concentrations [145]. Overall, these behaviors are consistent with the polyampholyte theory and with the hypothesis that LLPS is driven by the annealing of opposite-sign charges or by cation–π interactions.

In contrast with the three examples above, the N-terminal prion-like domain of galectin-3 undergoes LLPS only when the NaCl concentration is increased above 600 mM, with LLPS being driven by π–π interactions between aromatic residues [146].

Examples also exist where salt, namely NaCl, does not exert any significant impact on LLPS, suggesting that the formation of coacervates relies on hydrophobic interactions. An illustrative example is provided by PNT3, a viral protein region that undergoes LLPS with concomitant formation of amyloid-like fibrils [147]. Although this protein is classified as a strong polyampholyte (FCR = 0.364, |NCPR| = 0.164), NaCl does not affect its ability to form aggregates (at least up to 300 mM). In support for the hypothesis of the involvement of π–π interactions, the most amyloidogenic region contains three contiguous tyrosine residues whose replacement with alanine residues abrogates fibrillation [147]. Finally, we can cite the case of γ44-gliadin, a wheat storage protein with an intrinsically disordered domain that undergoes LLPS in a salt-dependent manner in spite of its very weakly charged nature (FCR = 0.04 and NCPR = 0) [148]. In particular, increasing NaCl concentrations were found to lead to a drastic decrease in the number of droplets and to an increase of γ44-gliadin saturation concentrations, corresponding to equilibrium concentrations above which phase separation occurs in in vitro experiments. This behavior argues for a contribution of electrostatic interactions in the formation of γ44-gliadin liquid-like droplets. Since the few charges are mainly located in the C-terminal domain, the authors proposed that this unequal charge distribution along the sequence likely promotes directional interactions: Aromatic residues of the N-terminal domains and positively charged residues of the C-terminal ones could participate in LLPS by π–cation interactions, as already reported [134,149,150].

The relentless tug of war between electrostatics and the hydrophobic effect is also a determinant for the link between aggregation and LLPS. To cite an instance, a connection between aggregation and LLPS of tau protein has for a long time been suggested [151,152,153] and recently ruled out [154]. For tau protein, LLPS is driven by complex coacervation mechanisms, dominated by long-range multivalent electrostatic attractions [155]. Instead, the amyloidogenic pathway seems driven by hydrophobic interactions [154]. In line with the behavior of tau, in the case of hnRNPDL, LLPS exerts a protective role against fibril formation [144]. On the contrary, when LLPS is triggered in vitro under high-salt concentrations, thereby becoming partially driven by hydrophobic contacts, a direct correlation with increased amyloid propensity is observed, suggesting that this two phenomena not only could occur under coinciding conditions but could also positively influence one another [154].

Although we can try to rationalize these complex responses, we are still far from acquiring the ability to predict them. The emerging scenario from the available literature data is that the effect of salt on LLPS is poorly predictable and highly protein dependent, analogously to its impact on protein fibrillation (see Section 4). This lack of a clear trend may depend on the double role of charged residues in the so-called architecture of “stickers” and “spacers” [141,156,157]. Stickers are protein motifs or domains reversibly interacting with other protein molecules or nucleic acids, thus creating coacervate networks. Spacers are located between stickers and preferentially interact with solvent molecules, acting as a scaffold [141,156,157]. In contrast with aromatic residues, which are definitely enriched in stickers’ elements, charged residues could play a role either in stickers, by electrostatic attractions, or in spacer regions, by conferring solubility and flexibility to the scaffold itself. This ambiguity can also entail the difficulty of designing electrostatically driven LLPS models with predictable and controllable behavior [142].

**Figure 5:**
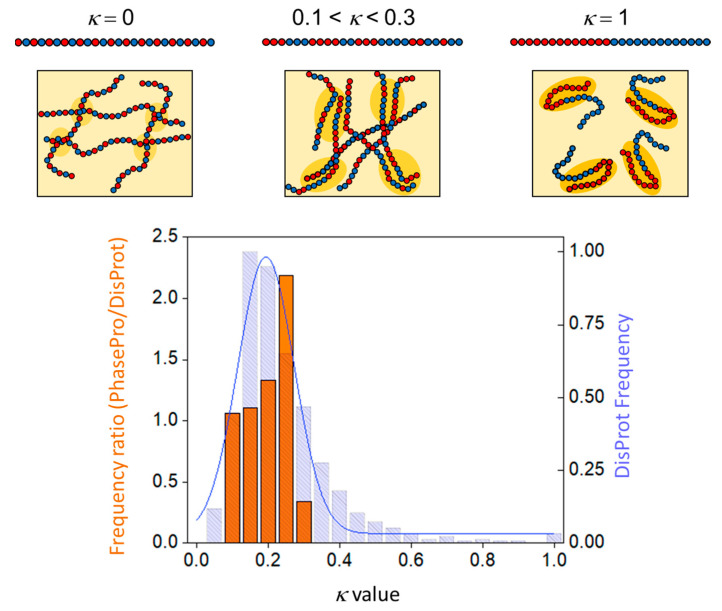
Charge decoration and propensity to undergo electrostatically driven phase separation. (**Top**) Lys-Glu sequences of different κ values, with K and E residues in red and blue, respectively. Charge distribution is related to protein compactness and phase separation (square boxes). κ = 0: attractions within and among polyampholytes lacking long same-charge clusters are weak. These chains are overall expanded and show weak scarcely cooperative inter-chain interactions, as symbolized by small ovals in pale yellow (scheme inspired by [46]). 0.1< κ < 0.3: most favorable inter-chain interactions among chains presenting blocks of segregated charges. κ = 1: complete, or almost complete, charge segregation favors strong intra-chain interactions (dark yellow areas) that, reflecting monomolecular events, efficiently out compete inter-chain attractions. (**Bottom**) IDP/IDR propensity to undergo electrostatically driven LLPS as a function of κ values. The orange histogram shows the ratio between PhasePro (subset of electrostatically driven phase separation) and DisProt k-class frequencies [159] (left vertical axis). The κ-class frequencies from DisProt are shown as the shadowed blue bar histogram (right-hand vertical axis). Sequences from the PhaSePro database were manually retrieved and further analyzed for their level of disorder by IUPred [161]. Only regions with an overall disorder level higher than 0.6 (in a scale 0–1) were used to compute κ values. The latter were calculated through CIDER webserver (http://pappulab.wustl.edu/CIDER/, [11]). DisProt entries were filtered by discarding sequences shorter than 20 amino acids or devoid of charged residues (FCR = 0) and redundant sequences. Two sequences were considered redundant if they were associated to the same DisProt_ID and if the start (residues 1–20) and the end (last 20 residues) of the two compared regions are respectively comparable.

**Figure A2:**
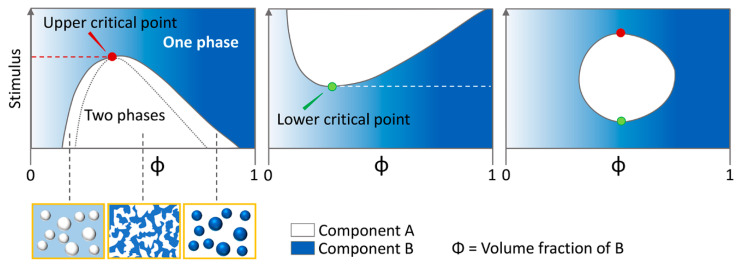
A two-component mixture can give rise to a single-phase well-mixed system, or to a two-phase system, in which the two components are separated. The separation into two phases can occur below or above a critical value of temperature, pH, ionic strength, etc. The diagram illustrates the coexistence curve (binodal) of three reference systems in response to a generic stimulus. (**Left side**): the transition can occur below a threshold point, which is therefore called the upper critical point (UCP). Phase separation is only observed upon a rate-limiting nucleation event, under conditions lying in between the binodal (indicated as solid line) and spinodal curves (indicated as dotted line). Once the spinodal curve is crossed, nucleation becomes dispensable and phase separation spontaneously occurs by spinodal decomposition. The pictorial schemes in the yellow boxes show spinodal decomposition (in the middle) and de-mixed states of components A and B (left and right); (**Middle**): phase separation occurs beyond the lower critical point (LCP); (**Right side**): this system features both UCP and LCP behavior.

An even smaller number of studies deal with the role of charge distribution in LLPS. Coacervation was found to strongly depend on the segregation of opposite charges in the Ddx4^N^ and Nephrin intracellular domain, which also a share similar value of κ (= 0.237 and 0.217, respectively) [134]. For instance, LLPS was suppressed by attenuating charge segregation in Ddx4^N^ in a permutant with κ = 0.053 [134]. A systematic analysis of the relationship between LLPS propensity and the linear pattern of charge distribution, namely the values of κ, has been addressed by computationally predicting the phase diagrams of (Glu-Lys)_25_ by a random-phase approximation approach [46]. Overall, the system shows a binodal curve with an upper critical temperature of coacervation (see Figure A2), which correlates well with κ. Since Rg decreases with increasing κ, a power law linking *R_g_* to the critical temperature (CT) holds as well [46]. Thus, the more compact the conformation, the higher the critical temperature, which is overall consistent with experimental evidence [134,158]. We propose, however, the existence of a “compaction threshold”, above which the dependence of CT on κ is inverted. That is, compaction would promote phase separation up to a certain limit, beyond which highly collapsed conformations would instead disfavor inter-chain interactions. In our hypothesis, IDPs/IDRs with low-to-moderate κ values display a higher propensity to undergo LLPS, while those containing interspersed charged residues (κ~0), as well as those with highly segregated charges (κ~1), present a lower coacervation propensity (Figure 5, top panel). To test this hypothesis, IDRs from the PhaSePro database [159] undergoing electrostatically driven phase separation were analyzed herein to extract a κ-value distribution. Each frequency class was compared with that of IDRs from the entire DisProt database. As shown in Figure 5, the frequency of sequences with 0.2 < κ < 0.25 in PhasePro is double compared to DisProt. By contrast, the frequency of sequences with 0.25 < κ < 0.3 is less than half (Figure 5, bottom panel). These data indicate that IDRs undergoing electrostatically driven phase separation have κ values mostly comprised between 0.2 and 0.25. Such κ values, as in the case of Ddx4 [66,160] and NICD [158], are those that probably allow to better exclude the solvent and favor inter-chain interactions. Nonetheless, the still limited size of PhaSePro (28 entries of IDPs/IDRs undergoing electrostatically driven phase separation at the time of this analysis) points to the need of addressing this issue in a more systematic way in the future, so as to be able to confirm the existence of an optimal value of κ favoring phase separation and possibly draw general conclusions. Our hypothesis is in line with a recent work indicating how critically important the patterning of sticking elements is, with the implication that too many sticky elements may hamper coacervation [141].

An obvious limitation in LLPS studies is that in vitro experiments cannot reproduce the complexity of the intracellular environment. Therefore, the picture needs to be further detailed, bearing in mind the peculiar features of the cellular context, in addition to simplified molecular models.

## 6. Conclusions

Today more than ever, knowledge concerning the role of electrostatics on the structure and function of IDPs/IDRs benefits from theoretical, experimental, and heuristic contributions from the fields of polymer physics and computational science. Tapping into such concepts has become imperative albeit challenging, as already pondered more than 20 years ago by one of the fathers of modern molecular biology [162]. Useful descriptors of IDP/IDR charge density and patterning, such as NCPR, κ, and *SCD*, arise from this very productive crosstalk between polymer theory, biophysics, computational simulations, and protein science, and contribute to deciphering the hidden structural code of IDPs/IDRs. The unfolded states of proteins have long been recognized as crucial models to interpret relevant biological processes, such as protein folding, membrane translocation, and stability, aided by the theoretical framework of statistical and polymer physics [163]. In this regard, IDPs/IDRs represent an interesting experimental model, as they offer significantly populated unfolded states in the absence of denaturants. One useful aspect resides in their remarkable designability and stimuli responsiveness. The high designability of disordered proteins allows for functional remodeling and modification of entire biological networks [164,165].We have learnt that such a reshaping can be obtained by tuning simple sequence parameters, such as the fraction of positive and negative charges and their clustering. The abundance and distribution of charged residues can confer specific sensitivity to changes in the environment, i.e., pH, ionic strength and ligands [56,83]. This plasticity is likely one of the reasons of their evolutionary success in regulatory networks. An interdisciplinary approach is essential for (i) a deeper understanding of the molecular mechanisms underlying physiological and pathological events, and (ii) translating our knowledge on polymers physics into de novo design of polypeptides with the desired properties of compactness, fibrillation, and phase separation, envisaging expectedly impactful biotechnological applications. Our knowledge of polymer physics does not yet allow to fully understand and especially control these events, particularly concerning phase separation, fibrillation, and their connection. This is partly due to the fact that this research field is still in its infancy. This area of research will benefit from the growth of dedicated databases, such as AmyPro [166], CPAD [167], DrLLPS [168], or PhasePro [159], and from data analysis through advanced data-mining tools, which will also become increasingly available in the future.

## Figures and Tables

**Table 1:** Examples of intrinsically disordered proteins (IDPs) and protein regions (IDRs) belonging to the distinct regions of the Das-Pappu’s conformational phase diagram.

Protein Class	FCR	NCPR	Representative Proteins	References
R1, Globules	<0.25	<0.25	α-Synuclein (residues 1–100)	[11]
R2, Globules and coils	0.25 ≤ FCR ≤ 0.35	≤0.35	Tau repeat domain	[11]
R3, Polyampholyte coils or hairpins	>0.35	≤0.35	LEA_4 proteins ^1^, NSP1 ^2^	[38]
R4, Polyelectrolytic semi-flexible rods or coils	>0.35	>0.35	Synthetic polyE and polyK; protamines, NP1 ^3^; RAG2 ^4^	[29,32]

^1^ Late Embryogenesis Abundant proteins from *Arabidopsis thaliana*; ^2^ nucleoporin Nsp1 (UniProt ID: P14907) of *S. cerevisiae*; ^3^ NP1 (UniProt ID: O13030), residues 5–24 of *Cynops pyrrhogaster* protamine 1; ^4^ RAG2 (UniProt ID: P21784), residues 392–411 (‘acidic hinge’) of *Cricetulus griseus* V(D)J recombination-activating protein 2.

**Figure A1:**
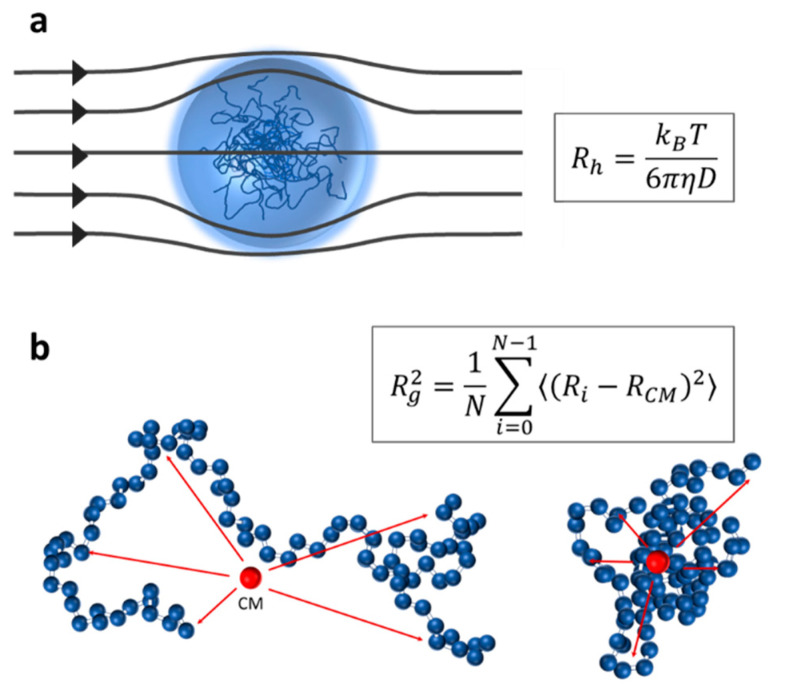
(**a**) Schematic representation and definition of *R_h_* (Figure adapted from [171]). (**b**) Schematic representation and definition of *R_g_* showing two conformations sampled from the ensemble of an ideal polymer, a freely jointed chain of 85 subunits, highlighting the position of their mass center (CM, at position *R_CM_*) and its distance from given subunits at generic position R_i_. A bias toward compact or extended conformations, reshaping the conformational ensemble, results in different average distances between CM and each subunit and, hence, different <R_g_> values.

## Abbreviations

Cryo-EM	Cryo-electron microscopy
CT	Critical temperature
FCR	Fraction of charged residue
GdmCl	Guanidinium chloride
IDP	Intrinsically disordered protein
IDR	Intrinsically disordered region
LCP	Lower critical point
LLPS	Liquid-liquid phase separation
MC	Monte Carlo (simulation)
NCPR	Net charge per residue
NMR	Nuclear magnetic resonance (spectroscopy)
PNT	N-terminus moiety of measles virus phosphoprotein
ProTα	C-terminal domain of Protα
PrP	Human prion protein
RAM	RBP-Jk-associated-molecule (region)
*R_DA_*	Donor-acceptor distance
*R_g_*	Radius of gyration;
*R_h_*	Hydrodynamic radius
rNFH-SA	Disordered region from the heavy subunit of the rat neurofilament complex
SAXS	Small angle X-ray scattering (spectroscopy)
*SCD*	Sequence charge decoration
SEC	Size-exclusion chromatography
smFRET	Single-molecule Förster resonance energy transfer
α-Syn	α-Synuclein
UCP	Upper critical point

## Appendix A. Parameters of Polymer Dimension: Hydrodynamic Radius, Radius of Gyration, and Their Relationships

According to the IUPAC recommendation, the dimensions of linear flexible macromolecules are usually expressed as the ensemble averages of the end-to-end distance, S, and radius of gyration *R_g_* [169]. Notably, a pronounced decoupling between *R_g_* and S has been observed, especially for heteropolymers, in both globule and coil-like states, probably due to the chemical heterogeneity of interactions [37]. Along *R_g_* and *S*, the *R_h_* can also return a reasonably adequate representation of IDP average dimensions and coil-globule transitions. The *R_g_* and *R_h_* parameters are measurable by biochemical/biophysical techniques and suitable to investigate the impact of sequence determinants on IDP/IDR conformation. As physical principles underlying *R_g_* and *R_h_* are distinct, they report on slightly different protein features.

The *R_h_* is the radius of an idealized sphere having the same diffusion coefficient as the molecule of interest (Figure A1a). It can be calculated according to the Stokes–Einstein relation in Equation (A1), where *k_B_* is the Boltzmann constant, *T* is the temperature, *η* is the viscosity coefficient of the medium, and *D* is the translational diffusion coefficient:

(A1) $$R_{h}=\frac{k_{B}T}{6\pi \eta D}.$$

*R_h_* can be experimentally measured by SEC, pulsed-field-gradient NMR, dynamic light scattering, and analytical ultracentrifugation.

The *R**_g_* is numerically assimilable to *R**_h_*, yet conceptually different and specifically used in polymer physics to describe the dimension of a polymer chain (Figure A1b). It can be measured through static light scattering, small angle neutron- and X-ray scattering, or calculated from simulated conformational ensembles. For a polymer composed of *N* subunits, *R**_g_* is defined by Equation (A2):

(A2) $$R_{g}^{2}=\frac{1}{N+1}\sum_{i=0}^{N}{\langle (R_{i}-R_{CM})}^{2}\rangle ,$$

where *R**_i_* is the vector indicating the position of any subunit and *R**_CM_* is the vector indicating the position of the polymer mass center [170]. *R**_g_* is averaged over the polymer ensemble, as denoted by the angular brackets 〈…〉 (Figure A1b). Notably, *R**_g_* is also proportional to the end-to-end distance *S*, according to the following equation:

*R_g_^2^* = *S*^2^/6. (A3)

For the sake of completeness, *S* is given by:

(A4) $$S^{2}={\langle (R_{N}-R_{0})}^{2}\rangle ,$$

where *S* is the average end-to-end distance between the position of the first (*R*_0_) and the latter subunit (*R_N_*) over the ensemble.

For an ideal polymer whose residue units do not interact with each other, i.e., a freely jointed chain, *R_g_* is given by:

(A5) $$R_{g}=\frac{1}{\sqrt{6}}\sqrt{N}a,$$

where *a* is dependent on the polymer stiffness and can vary over orders of magnitude. The scaling law of Equation (A5) could be re-written in more general terms as Equation (A6), to relate both *R**_g_* and *R**_h_* to chain length, i.e., to *N*:

(A6) $$R_{xs}=R_{0xs}N^{\nu_{xs}},$$

where *R*_0_ is the value for the compact state, in a given solvent, for structures where the volume scales linearly with the number of subunits; *x* = (*g*, *h*) determines whether the relationship refers to *R_g_* or *R_h_*; and *s* = (folded, unfolded, IDP) refers to the specific conformational class. It can be observed that the scaling law has not yet been declined for the different categories of IDPs. Empirically determined values for these parameters reveal that the scaling exponents v can vary from 0.33, for folded proteins, to 0.6 for disordered ones [171].

An empirical linear relationship between the *R_g_* of a simulated conformational ensemble and its *R_h_* was derived by Choy and collaborators [172]:

(A7) $$R_{g}/R_{h}=aR_{g}+b.$$

Although a roughly linear relationship exists for each protein, the values of *a* and *b* are different and vary with the protein length. This issue was subsequently addressed by Nygaard and co-workers [171], through an empirical method based on *R**_g_* and *R**_h_* data for 100 conformations of 30 IDPs or IDP-like peptides:

(A8) $$\frac{Rg}{R_{n}}\left(N,R_{g}\right)=\frac{\alpha_{1}\left(R_{g}-a_{2}N^{0.33}\right)}{N^{0.60}-N^{0.33}}+\alpha_{3},$$

where *α*_1_ = (0.216 ± 0.001) Å^−1^, *α*_2_ = (4.06 ± 0.02) Å, and *α*_3_ = (0.821±0.002), obtained as the best-fit values for empirical data from calculations/simulations.

## Appendix B. Protein Fibrillation and Peptide Self-Assembly

The misfolding of proteins and peptides can lead to supramolecular assembly and ordered amyloid-type fibrils [173], as typically observed in neurodegenerative disorders. This transition results in a cross-β (core) structure [173] and can be triggered by different physicochemical stimuli (e.g., temperature, Coulomb interaction, pH, metal ions, and chemical additives). Chain segments embedded within the cross-β core generally possess hydrophobic clusters, few charged residues, and patterns of alternating hydrophobic and hydrophilic amino acids, as well as an intrinsic β-sheet propensity [173]. Amyloid fibrils maximize the number of hydrogen bonds and hydrophobic interactions along the fibril axis, which generally is achieved through a parallel in-register arrangement of strands. Such an extended hydrogen-bonded β-sheet imparts mechanical strength and stability to amyloid fibrils, regardless of the folded or disordered nature of the native state [174,175,176]. A continuum of accessible pathways for amyloid conversion has been suggested, dependent on the environmental conditions, sequence, and conformational state of the amyloidogenic monomer [173,177,178,179]. Such alternative mechanisms could converge into the formation of growth-competent nuclei or aggregates, which support conversion into amyloid-like oligomers and eventually fibrils. This variety of fibril formation pathways could concern even a same polypeptide chain, resulting in polymorphism of the mature fibrils.

A way to tackle the extraordinary complexity of these structural transitions consists in investigating and manipulating self-assembling peptides (16–20 residues). Self-assembly refers to the association of two or more molecules, giving rise to ordered nanostructures (e.g., nanofibers, nanoribbons, nanotubes, or vesicles), from a few nanometer to hundreds of micron size. Such materials attract enormous interest as being bio-compatible and suitable to green technology and biomedical applications. Their formation is due to the synergistic and cooperative effect of various intramolecular and intermolecular non-covalent interactions, including hydrogen bonding, π–π stacking, electrostatic, hydrophobic and van der Waals interactions, as well as metal–ion coordination, which are also relevant for the formation of amyloid fibrils [180]. In line with the focus of this review, some examples of electrostatically driven self-assembly are described here. Peptide nanostructures based on electrostatic interactions have been reviewed [181]. The 20-mer MAX1 is mainly composed of Val and Lys residues (pI: 10.85) and reversibly responds to pH with gelification [182]. Under basic conditions, due to the neutralization of Lys side chains, the peptide folds into an amphiphilic β-hairpin with one face lined by hydrophobic Val residues and the other face exposing hydrophilic Lys residues. Self-assembly then occurs between hairpins both laterally, via H-bond formation, and facially, by hydrophobic association of the valine-rich faces of the folded peptide [182].

Another example is represented by the group of ionic self-complementary peptides, with a net charge that is almost zero at neutral pH and special amphiphilic features. In particular, RADA (RADA4 or RADA16) has been widely studied as it forms relatively regular nanofibers giving rise to a hydrogel exploitable for cell culturing, encapsulation, and molecule delivery in vivo. One side of the RADA4 monomer is thought to consist predominantly of nonpolar hydrophobic Ala (A), and the other side of alternating oppositely charged amino acids, namely Arg (R) and Asp (D). Recent publications suggest that the surface net charge is a crucial physicochemical parameter in protein aggregation [183,184]. The latter work, based on CD analysis and metadynamics simulations, concludes that RADA16 fibrillation could be easily modulated by pH and ionic strength. In particular, fibril formation is promoted at pH~pI, when a low net charge is achieved. This is due to poor electrostatic repulsions, favoring intermolecular interactions, and to the peptide conformation. Indeed, at this pH, RADA16 is predicted to have a β-hairpin conformation, promoting fibril formation. Ionic strength can promote fibrillation even at pH far from pI and in the presence of a large number of uncompensated charges, by shielding effects. This study highlights the main aspects that have to be taken into consideration when describing protein fibrillation, i.e., the effect of polymer charges and solution charges on conformation, aggregation propensity, and fibril shape. These issues are addressed in more detail in Section 4 of the main text.

## Appendix C. Liquid–Liquid Phase Separation

In polymer chemistry, liquid–liquid phase separation (LLPS) occurs whenever polymer self-interactions prevail over polymer–solvent ones (poor solvent conditions), defining two immiscible phases, a low-concentration diluted phase and a high-concentration condensed one [128]. Figure A2 illustrates this concept referring to three kinds of stimuli-sensitive systems. Both phases retain liquid-like properties and the same chemical potential, thus impeding any net diffusive flux between them, yet permitting the rapid exchange of single molecules [128]. In cellular protoplasm, phase separation could account for the formation of proteinaceous, membraneless compartments assimilable to organelles, employed to control chemical reactions (specificity, inhibition, kinetics), store biomolecules, sequester damaging factors, and enhance signal transduction [129]. Such biomolecular condensates are optically resolvable as micron-sized, spherical, and deformable coacervates [129,150] localized either in the cytoplasm or in the nucleus. Among others, we can list, in the nucleus, nucleoli [185,186], Cajal bodies [187], PML (promyelocytic leukemia) nuclear bodies [188,189], and nuclear speckles [190]; and in the cytoplasm, P bodies, stress, and germ granules [191].

Multivalent molecules, such as IDPs and modular multi-domain proteins, elicit the formation of either large homogeneous or heterogeneous complexes, reducing macromolecule solvation preceding phase separation. All kinds of weak and non-specific interactions can contribute to such adhesive contacts. Specific involvement in coacervation has been documented for cation–π contacts, between positively charged residues and π electrons in aromatic residues, π–π interactions between aromatic rings, electrostatic attractions, and dipolar forces [128,136], along with hydrogen bonding [192] and hydrophobic interactions [193]. Poly-ions, as well as RNA or ssDNA, can seed LLPS by favoring the interactions of RNA with the RNA-binding domain [130] or RNA base pairing [129]. The consequent extent of interactions and spatial ordering within the droplet dictates its state of matter, which could be simply liquid, liquid-crystalline, liquid-gel, semi-crystalline-solid, crystalline-solid, solid-gel, or solid [129]. In particular, IDP-based liquid droplets likely undergo a progressive phase transition towards viscoelastic and then solid aggregates, eventually interrupting any material exchange with the cytoplasm. Several mechanisms could account for this hardening process, including the growth of amyloid fibrils, vitrification of amorphous aggregates, crosslinking, or simply entanglement of disordered chains [128]. Changes in the droplet physical properties are related to several functions [194,195]. For instance, LLPS and other forms of hardening may contribute to reduce the kinetics of reactions, benefiting from the increase in viscosity to slow the diffusion of molecules and thus reaction rates. This has led to envisage a biotechnological exploitation of the LLPS phenomenon in the field of enzymology and heterogeneous-phase catalysis [196]. However, the precise mechanisms underlying phase separation, and most of all their regulation, still remain to be fully elucidated.
